# Chemical Composition and Antioxidant and Antibacterial Activities of an Essential Oil Extracted from an Edible Seaweed, *Laminaria japonica* L.

**DOI:** 10.3390/molecules200712093

**Published:** 2015-07-02

**Authors:** Jayanta Kumar Patra, Gitishree Das, Kwang-Hyun Baek

**Affiliations:** School of Biotechnology, Yeungnam University, Gyeongsan, Gyeongbuk 712-749, Korea; E-Mails: jkpatra@ynu.ac.kr (J.K.P.); gitishreedas@gmail.com (G.D.)

**Keywords:** antibacterial, antioxidant, chemical composition, essential oil, *Laminaria japonica*, seaweed

## Abstract

*Laminaria japonica* L. is among the most commonly consumed seaweeds in northeast Asia. In the present study, *L. japonica* essential oil (LJEO) was extracted by microwave-hydrodistillation and analyzed by gas chromatography and mass spectroscopy. LJEO contained 21 volatile compounds, comprising 99.76% of the total volume of the essential oil, primarily tetradeconoic acid (51.75%), hexadecanoic acid (16.57%), (9*Z*,12*Z*)-9,12-Octadecadienoic acid (12.09%), and (9*Z*)-hexadec-9-enoic acid (9.25%). Evaluation of the antibacterial potential against three foodborne pathogens, *Bacillus cereus* ATCC 10876, *Escherichia coli* O157:H7 ATCC 43890, and *Staphylococcus aureus* ATCC 49444, revealed that LJEO at a concentration of 25 mg/paper disc exerted high antibacterial activity against *S. aureus* (11.5 ± 0.58 mm inhibition zone) and *B. cereus* (10.5 ± 0.57 mm inhibition zone), but no inhibition of *E. coli* O157:H7. LJEO also displayed DPPH (1,1-diphenyl-2-picrylhydrazyl) free radical scavenging activity (80.45%), superoxide anion scavenging activity (54.03%), and ABTS (2,2′-azino-bis(3-ethylbenzothiazoline-6-sulphonic acid) radical and hydroxyl radical scavenging at 500 µg/mL. Finally, LJEO showed high inhibition of lipid peroxidation with strong reducing power. In conclusion, LJEO from edible seaweed is an inexpensive but favorable resource with strong antibacterial capacity as well as free radical scavenging and antioxidant activity; therefore, it has the potential for use in the food, cosmetics, and pharmaceutical industries.

## 1. Introduction

Reactive oxygen species (ROS) and oxygen-centered free radicals are continuously produced in the human body as byproducts due to numerous biochemical, physiological, and metabolic activities that occur during normal physiological processes [1,2]. The adverse effects of ROS and free radicals are controlled by the body’s own antioxidant defense mechanisms; however, their overproduction causes oxidative damage to DNA, lipids, and proteins, leading to chronic diseases including cancer, diabetes, aging, atherosclerosis, inflammation, and other degenerative diseases [2,3]. Contamination of food products by oxidation and deterioration due to ROS and free radicals causes various diseases in consumers [4].

Increased consumption of dietary antioxidants or antioxidant-rich food products can help maintain a balance between oxidants and antioxidants in the body [5]. Many natural antioxidants with the potential to neutralize the adverse effect of harmful free radicals have been identified [6]. Moreover, various natural bioactive compounds and fatty acids have been tested for their potential application in the food, cosmetics, and pharmaceutical industries. Furthermore, many types of synthetic chemicals and preservatives have been tested in food preservation and processing to address the adverse effects of ROS [7,8]. However, excessive use of these synthetic drugs leads to potential health hazards and carcinogenicity, and thus rejection among consumers. Different types of food materials of animal and plant origin are always prone to spoilage and decaying phenomena caused by various microorganisms like bacteria and fungi [9,10,11]. The spoilage and decaying of food materials caused a heavy loss of food products and raw materials, leading to food poisoning and infections [9]. Alternatively, the use of synthetic antimicrobial agents in the processing of food materials also leads to health hazards and rejection among consumers. Accordingly, the cosmetics, food, and pharmaceutical industries have started to search for alternatives to synthetic antioxidants in natural plants owing to their rich phenolic molecules, which include flavonoids, alkaloids, and terpenoids [12]. The use of synthetic antioxidants in food can be reduced by natural antioxidants from plant sources. Accordingly, many plants and their products, particularly essential oils, have recently been tested for their potential antioxidant activity against adverse free radicals [6,13,14,15]. Essential oils extracted from plants have become a good source of natural antioxidants [16]. Moreover, there is growing interest in the use of marine organisms as a potential source of effective antimicrobial and antioxidant compounds [17]. Several seaweeds have been investigated for their potential antioxidant, antimicrobial, and antiviral activity [17,18,19]. The dichloromethane extract from the red seaweed *Ceramium rubrum* has been found to be most effective against *Yersinia ruckeri* and *Saprolegnia parasitica*, agents that cause diseases in salmonids [20]. Furthermore, various solvent extracts of the marine seaweed *Sargassum muticum* have also been found to be effective against human pathogens [21]. Accordingly, it is worth investigating seaweeds and their products for the presence of potent antioxidant compounds.

Edible seaweeds belonging to the classes Chlorophyceae, Phaeophyceae (particularly the order Laminariales), and Rhodophyceae are popularly consumed as food [22]. These seaweeds include *Porphyra tenera*, *Palmaria palmate*, *Hijikia fusiformis*, *Undaria pinnatifida*, and *Laminaria japonica*. The extracts of seaweed belonging to the order Laminariales possess various biological activities, including antibacterial, antioxidant, antifungal, and antiviral potential [23,24]. Among these, *L. japonica* is the most widely and commonly consumed seaweed in northeast Asian countries including the Korea, Japan, and China [23]. *L. japonica* has been included as a potent drug in traditional Chinese medicine [25] and cultivated commercially in coastal northeast Asian countries for the production of valuable polysaccharides used for agar and alginate [23]. It has been found that extracts of *L. japonica* have been classified as Generally Recognized as Safe (GRAS) to be used as flavoring agents [26] and are most commonly used as food in Asian countries like Japan, Korea and China [23], so it could be presumed that this component of *L. japonica* (essential oil) could also be suitable for food purposes and its applications in food processing and preservation are under study.

In the present study, we attempted to extract essential oil from dry sheets of edible *L. japonica* used to make soup, which are available at local markets in the Korea. The essential oil was characterized and its free radical scavenging activity, antioxidant potential, and antibacterial activity against three foodborne pathogens were evaluated.

## 2. Results and Discussion

### 2.1. Chemical Composition of LJEO

**Figure 1:**
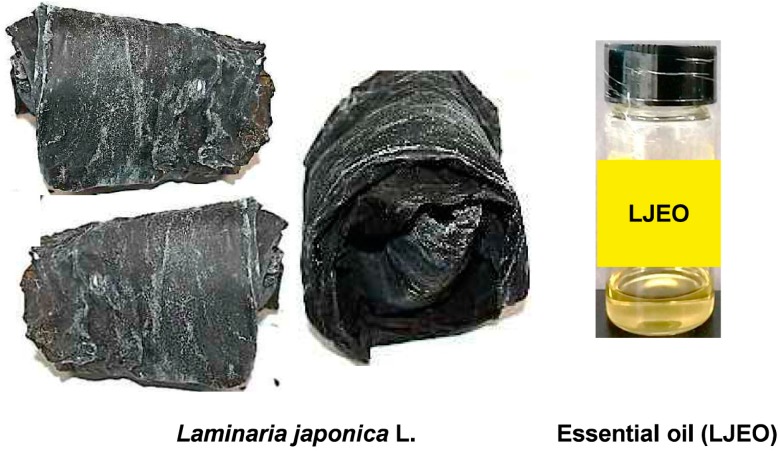
Dried sheet of *Laminaria japonica* L. and extracted essential oil.

**Figure 2:**
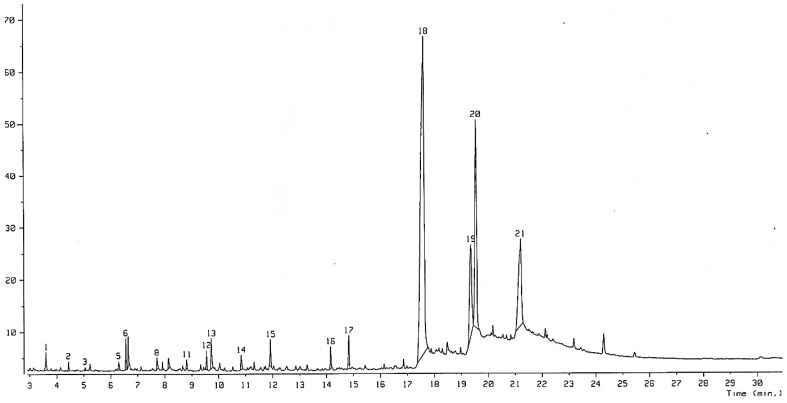
GC-MS analysis spectra with the identified number of compounds of *Laminaria japonica* L. essential oil (LJEO).

**Figure 3:**
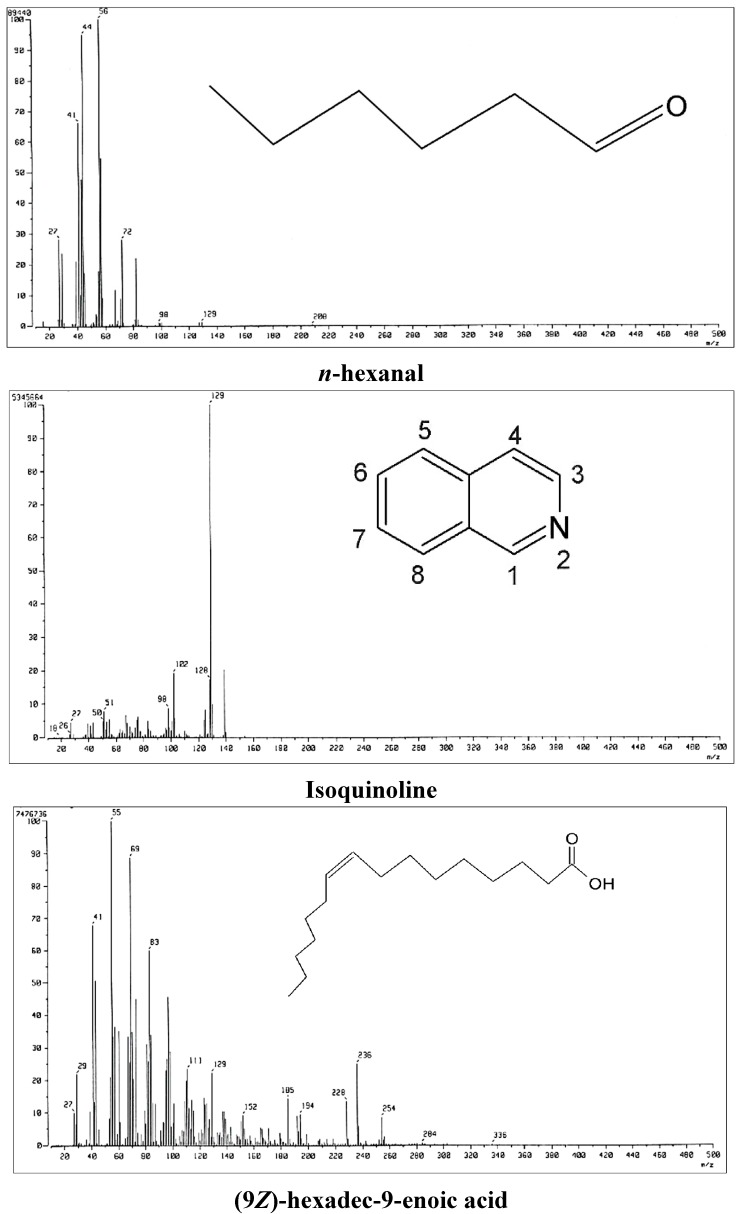

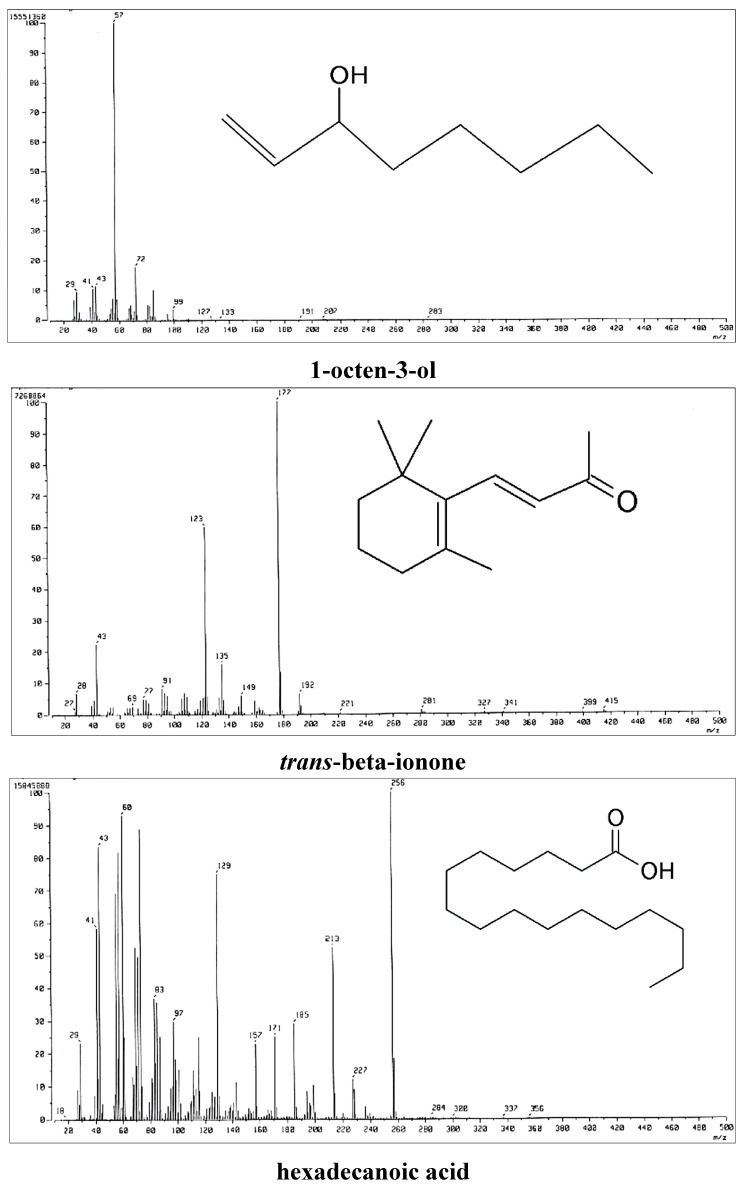

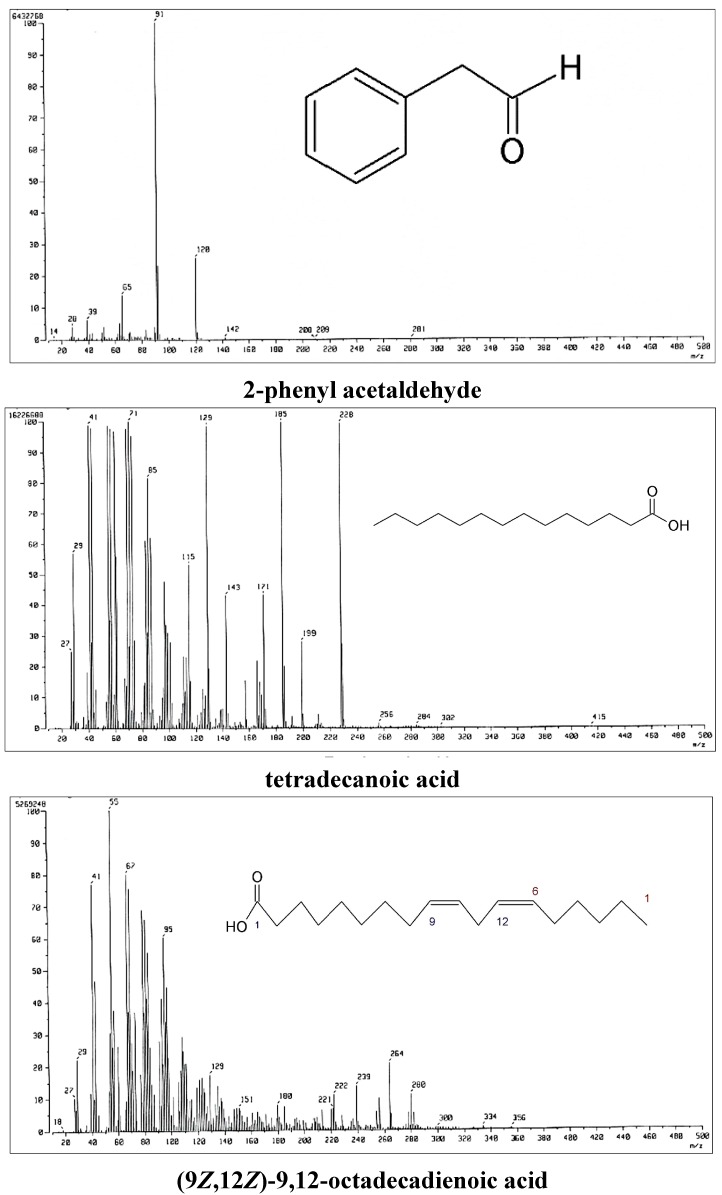
Profiles of mass spectra and chemical structures of important compounds present in *Laminaria japonica* L. essential oil (LJEO).

Complete knowledge of the chemical constituents of LJEO will facilitate synthesis of other chemicals and compounds for their potential applications in the cosmetics, food, and pharmaceutical sectors, as suggested by Iqbal [27]. The chemical composition of the extracted LJEO (Figure 1) was analyzed by GC-MS (Figure 2), which resulted in identification of 21 volatile compounds comprising 99.76% of the total volume. The main identified compounds were fatty acids (89.66%), ketones (3.43%), alcohols (2.68%), aldehydes (2.38%), monoterpenes (0.95%), and benzypyridine (0.66%). The dominant fatty acids consisted of tetradecanoic acid (51.75%), hexadecanoic acid (16.57%), (9*Z*, 12*Z*)-9,12-Octadecadienoic acid (12.09%), and (9*Z*)-hexadec-9-enoic acid (9.25%) (Table 1). The MS spectra and chemical structures of some important compounds are presented in Figure 3. Most of the compounds present in LJEO possess antifungal, insecticidal, antibacterial, antioxidant, and anti-inflammatory properties [28,29,30,31,32], while some compounds are used as flavoring agents or food additives [33,34]. Tetradecanoic acid (also known as myristic acid), which was found in high amounts, has been used as a flavoring agent and dietary supplement by food industries, as a component of facial creams and lotions, emulsifiers, and toiletries in the cosmetics industry; and as an ingredient for the development of drugs for the brain by pharmaceutical industries [35,36]. The presence of myristic acid in different types of edible kelp species has been reported as a major component along with hexadecanoic acid [37]; however, the number of identified compounds and their percentage composition varies with the present result, which might be due to the detection procedure and different types of species used for extraction of the essential oil and also the environmental conditions of where the seaweed species were collected. Hexadecanoic acid has been reported to possess anti-inflammatory, anticancer, and antioxidant properties [38,39,40,41,42]. (9*Z*,12*Z*)-9,12-Octadecadienoic acid (linoleic acid), present in a high quantity (12.09%), could be potentially used by the cosmetics industry for the production of beauty products because of its beneficial effects on the skin for application in antioxidant, anti-inflammatory, acne reductive, and moisture retentive products [43,44].

**Table 1:** Chemical constituents of *Laminaria japonica* L. essential oil (LJEO), based on GC-MS analysis.

Functional Group	No. ^*^	SI ^**^	RT ^***^	Compound ^#^	Composition (%)
Aldehyde	1	892	3.60	*n*-hexanal	0.63
	4	900	5.23	*n*-heptanal	0.19
	5	887	6.30	benzaldehyde	0.32
	8	812	7.71	2-phenyl acetaldehyde	0.47
	9	845	7.91	*trans*-2-octenal	0.21
	12	864	9.54	*trans*-2-nonenal	0.56
Ketone	3	874	5.05	Heptan-2-one	0.09
	6	825	6.56	1-octen-3-one	0.88
	11	594	8.81	Heptan-2-one	0.28
	16	751	14.16	*trans*-beta-ionone	0.78
	17	900	14.84	2(4H)-benzofuranone	1.40
Alcohol	2	884	4.43	2-hexen-1-ol	0.24
	7	882	6.65	1-octen-3-ol	0.85
	10	717	8.13	2-octen-1-ol	0.50
	13	824	9.73	trans-2-undecen-1-ol	1.33
Fatty acid	18	815	17.64	tetradecanoic acid (myristic acid)	51.75
	19	749	19.37	(9*Z*)-hexadec-9-enoic acid (palmitoleic acid)	9.25
	20	812	19.57	hexadecanoic acid (palmitic acid)	16.57
	21	624	21.22	(9*Z*,12*Z*)-9,12-Octadecadienoic acid (linoleic acid)	12.09
Benzypyridine	14	621	10.84	isoquinoline	0.66
Monoterpene	15	507	11.92	isocineole	0.95

^*^ No.: Compound number in order of elution; ^**^ International System of Units (SI values) library search purity value of compound; ^***^ RT-Retention time; ^#^ Compound-identified based on EI-MS.

### 2.2. Antibacterial Activity of LJEO against Foodborne Pathogens

The antibacterial activity of LJEO against three foodborne pathogenic bacteria is presented in Table 2. LJEO exerted antibacterial activity against *S. aureus* ATCC 49444 and *B. cereus* ATCC 10876, as indicated by zones of inhibition of 11.5 and 10.5 mm, respectively (Table 2); however, it exerted no antibacterial effect on *E. coli* O157:H7 ATCC 43890. The positive control (kanamycin) also exerted antibacterial activity, generating zones of inhibition of 12–13 mm against all tested pathogens (Table 2), whereas the negative control [5% dimethylsulphoxide (DMSO)] exerted no inhibitory activity. The antibacterial effects of LJEO against *S. aureus* ATCC 49444 and *B. cereus* ATCC 10876 might be due to the nature of the bacterial outer cell wall, which facilitates penetration of LJEO into the bacterial cell, eventually causing death [45]. The outer cell wall of Gram-positive bacteria lacks an outer membrane but is surrounded by thick peptidoglycan layers that might have facilitated the easy penetration of the LJEO into the bacterium, causing destruction of the bacterial cell. In contrast, the Gram-negative bacteria are surrounded by a thick outer membrane containing lipopolysaccharide and a thin peptidoglycan cell wall [46]; therefore, this thicker outer membrane might not be permeable to LJEO and that was why LJEO did not show any antibacterial effect against the Gram-negative *E. coli* O157:H7 ATCC 43890 but exerted potent antibacterial effect against the Gram-positive *S. aureus* ATCC 49444 and *B. cereus* ATCC 10876. The minimum inhibitory concentration (MIC) and minimum bactericidal concentration (MBC) of LJEO for the two controlled pathogens were both 25 mg/mL (Table 2); this data was similar to the results obtained from the essential oils of many seaweeds and their products, which had antimicrobial activities against different pathogenic microorganisms [11,47,48,49]. The food industry primarily uses different types of essential oils from various sources as flavoring agents; however, their potential application can work as an interesting source of natural antimicrobial compounds for food preservation that act effectively against the foodborne pathogenic microorganisms [50]. The antibacterial activity of LJEO might be attributed to the presence of various antibacterial compounds, including *n*-hexanal, phenyl acetaldehyde, isoquinoline, and tetradecanoic acid [28,29,30,50].

**Table 2:** Antibacterial activity of *Laminaria japonica* L. essential oil (LJEO) at 25 mg/disc against foodborne pathogenic bacteria.

Foodborne Pathogens	LJEO ^#^	Positive Control ^*,#^	Negative Control ^**,#^	MIC	MBC
*S. aureus* ATCC 49444	11.5 ± 0.58 ^bc^	13.0 ± 2.64 ^a^	0 ± 0	25	25
*B. cereus* ATCC 10876	10.5 ± 0.57 ^c^	12.0 ± 1.52 ^ab^	0 ± 0	25	25
*E. coli* O157:H7 ATCC 43890	0 ± 0 ^d^	12.0 ± 2.08 ^ab^	0 ± 0	0	0

* Kanamycin at 40 µg/disc as the positive control; ** 5% DMSO/disc as the negative control; **^#^** Diameter of inhibition zone in mm of three independent replicates expressed as the mean ± standard deviation; values with different superscript letters in LJEO and positive control are significantly different at *p* < 0.05.

### 2.3. Free Radical Scavenging and Antioxidant Activity of LJEO

Due to the potential for different types of antioxidant compounds in LJEO, *in vitro* antioxidant screening methods were applied to determine the antioxidant effects of LJEO. The free radical scavenging potential of LJEO was assayed by DPPH radical scavenging assay, ABTS radical scavenging assay, hydroxyl radical scavenging assay, and superoxide anion scavenging assay.

**Figure 4:**
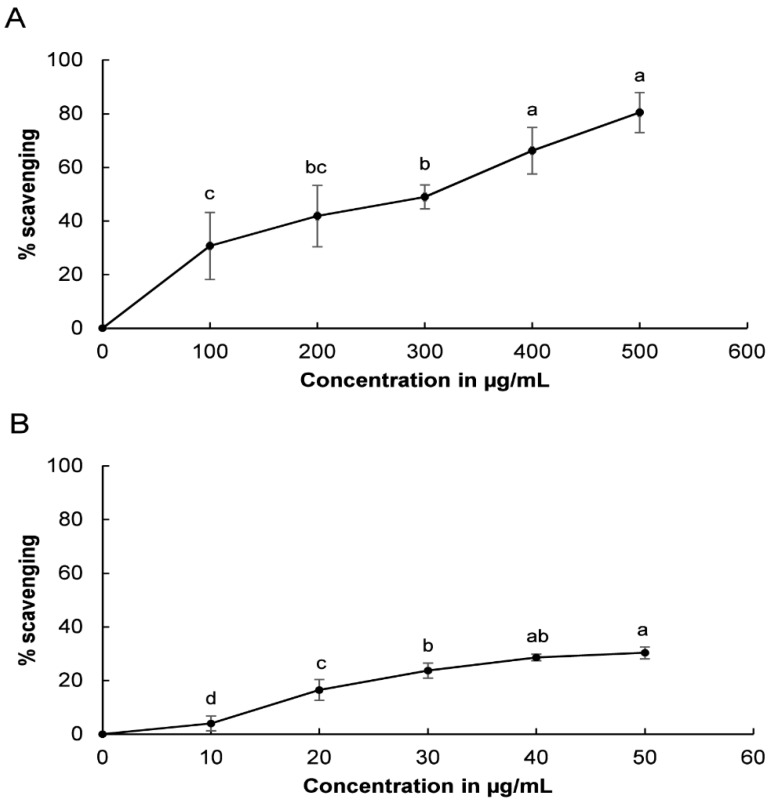
DPPH scavenging potential of (**A**) LJEO and (**B**) BHT as a reference. The results are the mean value of three independent replicates; values with different superscript letters in each section are significantly different at *p* < 0.05. The superscript letter “ab” or “bc” on a value shows that the value did not differ significantly from other values with the superscript letter “a” or “b” and “b” or “c”.

The DPPH radical scavenging potential of LJEO is presented in Figure 4. LJEO and BHT showed radical scavenging potential of 80.45% at 500 µg/mL and 30.31% inhibition at 50 µg/mL, respectively (Figure 4). Both LJEO and BHT increased DPPH radical scavenging activity with higher concentrations. DPPH is a standard free radical that is neutralized by the donation of hydrogen ions or electrons from an external source [51,52]. In the present investigation, the DPPH radical was neutralized by addition of LJEO to the reaction mixture, which was confirmed by the change in color of the solution from purple to yellow. This change in color might be attributed to the donation of hydrogen ions or electrons by LJEO to neutralize DPPH free radicals. Furthermore, the effects of LJEO on DPPH free radicals could scavenge peroxy radicals, which propagate lipid peroxidation by terminating the chain reaction [14,53]. The high DPPH free radical scavenging potential of LJEO may be due to its rich content of fatty acids, including hexadeconic acid, (9*Z*)-hexadec-9-enoic acid, and tetradecanoic acid, which are effective DPPH free radical scavengers [36,38,39,40,41].

The ABTS free radical scavenging activity of LJEO and the reference compound, BHT, is shown in Table 3. The IC_50_ of LJEO and BHT was 92.98 µg/mL and 19.65 µg/mL, respectively. The ABTS scavenging activity of LJEO also reflects its antioxidant potential, and the mechanism of action is similar to the scavenging of DPPH radicals by either donating a free electron or hydrogen atom to terminate the reaction [54,55].

**Table 3:** Free radical scavenging, inhibition of lipid peroxidation, and reducing power of *Laminaria japonica* L. essential oil (LJEO).

Extract	ABTS Radical Scavenging (IC_50_) ^*^	Hydroxyl Radical Scavenging (IC_50_) ^*^	Inhibition of Lipid Peroxidation (IC_50_) ^*^	Reducing Power (IC_0.5_) ^**^	Phenol Content (% Gallic Acid Equivalent)
LJEO	92.98 ± 1.69	158.18 ± 3.55	388.35 ± 3.07	89.35 ± 2.02	5.81 ± 0.60
BHT	19.65 ± 0.43	19.85 ± 1.35	35.16 ± 1.15	12.74 ± 1.41	

* IC_50_: Concentration of extract (µg/mL) showing 50% scavenging potential; ** IC_0.5_: Concentration of extract (µg/mL) showing 0.5 O.D. value at 700 nm. The results are the mean value of three independent replicates ± standard deviation.

The hydroxyl radical scavenging potential of LJEO and BHT is presented in Table 3. The IC_50_ value of LJEO and BHT to scavenge hydroxyl radicals was 158.18 µg/mL and 19.85 µg/mL, respectively. The hydroxyl radical, which is formed by the Fenton reaction in the presence of reduced transition metals and H_2_O_2_, is the most reactive and physiologically harmful free radical among the entire reduced dioxygen group, causing lipid peroxidation and even leading to cell damage and death [53,56]. The hydroxyl radical scavenging potential of LJEO could be an important indicator of its antioxidant potential that can act efficiently against oxidative damage caused by a wide range of biomolecules, such as lipids, proteins, sugars, and nucleotides [57,58]. Because it has a high hydroxyl radical scavenging potential, LJEO can be used as a food additive or food preservative.

**Figure 5:**
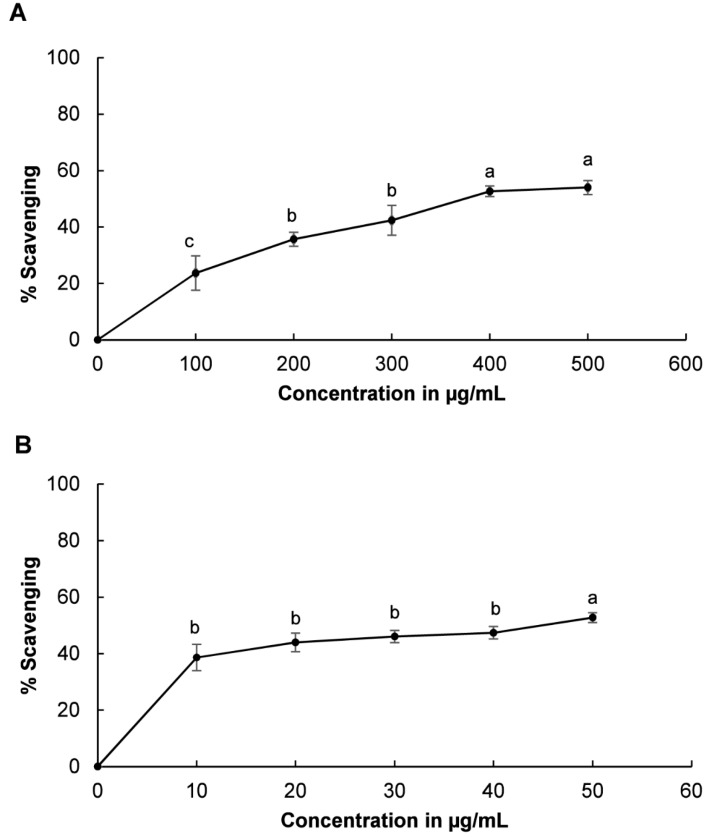
Superoxide scavenging potential of (**A**) LJEO and (**B**) BHT as a reference. The results are the mean value of three independent replicates; values with different superscript letters in each section are significantly different at *p* < 0.05.

Evaluation of the superoxide anion scavenging potential of LJEO and BHT revealed that both exerted superoxide radical scavenging activity in a concentration dependent manner, with LJEO and BHT showing potential of 53.88% at 500 µg/mL and 52.81% at 50 µg/mL, respectively (Figure 5). Under the influence of oxidative stress, the concentration of superoxide anions increased in the body, resulting in the generation of other ROS that cause serious damage to the body’s own macromolecules [59,60]. The superoxide scavenging potential of LJEO further confirmed its strong antioxidant potential, indicating it could be useful to the food, cosmetics, and pharmaceutical industries.

The antioxidant potential of LJEO was further investigated in terms of its inhibition of lipid peroxidation and reducing power. The potential for inhibition of lipid peroxidation of LJEO and the reference compound BHT is shown in Table 3. Both LJEO and BHT possessed moderate inhibition potential, with IC_50_ values of 388.35 µg/mL and 35.61 µg/mL, respectively. The deterioration of food items, decreasing their nutritive values and morphologies (color, texture, and appearance), is mainly caused by strong peroxidation of their lipid content [61]. The potential inhibitory effects of LJEO on lipid peroxidation make it a strong candidate for use as a food additive and preservative.

Both LJEO and BHT possessed strong reducing power, with IC_0.5_ values of 89.35 µg/mL and 12.74 µg/mL, respectively (Table 3). LJEO and BHT contained a concentration-dependent reducing potential. The total phenol content of LJEO was calculated to be 5.81% ± 0.60% of gallic acid equivalent based on the standard calibration curve (Table 3). Phenolic compounds have been known to be strong antioxidant compounds with the ability to scavenge free radicals [62,63]; therefore, the antioxidant potential of LJEO might be due to the presence of high levels of phenolic compounds [64,65].

## 3. Experimental Section

### 3.1. Extraction of Essential Oil from L. japonica and Analysis

Dry sheets of the edible seaweed, *L. japonica*, were purchased from a local market at Gyeongsan, Gyeongbuk, Korea (Figure 1). The seaweeds were cultivated and dried in Wando Island and distributed by the Wandodasima Company (Wando, Korea). The dried seaweed was crushed by hand into small pieces, weighed (250 g), and put into the glass chamber of the hydrodistillation unit, after which 3 L of distilled water was added. The extraction of *L. japonica* essential oil (LJEO) was then subjected to the hydrodistillation for up to 4 h using a microwave generating extraction machine manufactured by KMD Engineering (Yeoju, Korea) with a standard method described by Patra *et al.* [14]. The temperature of the extraction chamber was controlled by the thermo controller with a power capacity of 40 W and a frequency of 15 gkH. The extracted distillate was collected from the collecting nozzle in a conical flask attached to it. The extracted distillate was further mixed with dichloromethane solvent in a separating funnel, shaken vigorously for 15 min, and then held until the two layers settled completely. After 1–2 h, the lower layer of the separating funnel with LJEO was collected and concentrated using a rotary evaporator at a lower temperature of 40 °C. The extracted LJEO was dried over anhydrous sodium sulfate and kept at 4 °C in a tightly sealed glass vial until use.

### 3.2. GC-MS Analysis of LJEO

The chemical constituents of LJEO were analyzed by GC-MS (JMS 700 MStation, Jeol Ltd., Peabody, MA, USA) by the standard procedure [14]. The GC-MS system was equipped with an Agilent 6890N GC DB-5 MS fused silica capillary column of size 30 m × 0.25 mm i.d. and film thickness of 0.25 µm. For the detection of the volatile compounds, an electron ionization system with ionization energy of 70 eV was used. Helium gas was used in the machine as the carrier gas and was applied at a constant flow rate of 1 mL/min. The injector temperature and the temperature of the MS transfer line were set at 280 °C and 250 °C, respectively. At first, the temperature of the oven was maintained at 50 °C for 2 min; then the temperature was increased at a rate of 10 °C/min to 250 °C and held for 10 min at this temperature. An LJEO sample (1 µL of 100 times-diluted in methanol) was injected manually through the injector in splitless mode. The relative percentages of the constituents of LJEO were expressed as the calculated percentages by normalization of the peak area. The various volatile compounds present in LJEO were identified on the basis of the GC retention time on a DB-5 capillary column that are relative to the matching of mass spectra in the computer using the Wiley and National Institute of Standards and Technology libraries for the GC-MS system. The chemical structures of some important compounds with biological potential were drawn using the ACD Chemsketch software (ACD/ChemSketch Freeware, Advanced Chemistry Development, Inc., Toronto, ON, Canada).

### 3.3. Evaluation of Antibacterial Activity of LJEO

#### 3.3.1. Pathogenic Bacteria Used

Three different foodborne pathogenic bacteria were used in the present study to determine the antibacterial potential of LJEO. These include two Gram-positive bacteria, *Staphylococcus aureus* ATCC 49444 and *Bacillus cereus* ATCC 10876 and one Gram-negative bacterium, *Escherichia coli* O157:H7 ATCC 43890. These foodborne bacteria were obtained from the American Type Culture Collection (ATCC, Manassas, VA, USA) and maintained as glycerol stock at −80 °C.

#### 3.3.2. Antibacterial Evaluation

The antibacterial activity of LJEO was evaluated by the standard disk diffusion assay [66]. Prior to use, the three foodborne pathogenic bacteria were subcultured in nutrient broth (NB) media and diluted to a concentration of 10^7^ CFU/mL. LJEO was initially diluted with 0.5% Tween-80 to prepare a stock solution of 500 mg/mL and then further diluted with 5% DMSO to an appropriate concentration and sterilized by passing through a 0.22 µm nylon syringe filter (Chemco Scientific, South Korea). Paper discs with a diameter of 6 mm were impregnated with 25 mg/disc LJEO and then used to test the antibacterial potential of LJEO against the pathogens. DMSO (5%) was used as a negative control throughout the experiment, while kanamycin (40 µg/mL) was used as a positive control. Nutrient agar (NA) plates were spread with 100 µL of the bacterial culture in a sterile spreader using aseptic conditions. The paper disc with LJEO was placed over the NA plates and covered and left for 1 h for diffusion of the compound to the NA medium. The plates were then incubated at 37 °C for 24 h. The diameters of inhibition zones developed after 24 h of incubation at 37 °C were measured to determine the antibacterial potential of LJEO against the foodborne bacteria.

#### 3.3.3. Determination of Minimum Inhibitory Concentration (MIC) and Minimum Bactericidal Concentration (MBC)

The MIC and MBC of LJEO against foodborne bacteria were determined by the two-fold dilution method, with minor modification [67]. The active cultures of the bacteria were prepared by transferring the loopful of bacterial cells from the stock culture to the conical flasks containing NB media and incubating it at 37 °C for 24 h. The cultures were diluted with fresh media to obtain an optical density value of 107 CFU/mL at 600 nm. Different dilutions of the LJEO in the NB media were prepared in a 96-well microplate by the two-fold dilution method in the concentration range of 50, 25, 12.5, 6.25, 3.12, 1.56, and 0.78 mg/mL. Then 10 µL of each bacterial strain (10^7^ CFU/mL) was inoculated onto the microplates (SPL Life Sciences, Gyeonggi-do, Korea); the tests were performed in a volume of 100 µL. The plates were incubated at 37 °C for 24 h. The lowest concentrations of LJEO, which did not show any visual growth of the test organisms, were determined as the MICs, which were expressed in mg/mL. The MIC concentration and the next higher concentration of the sample were selected, spread on the NA plates, and incubated at 37 °C for 24 h; the concentration of LJEO, which did not show any growth of the microorganism on the NA plates, was determined as the MBC and expressed in mg/mL.

### 3.4. Free Radical Scavenging and Antioxidant Potential of LJEO

The free radical scavenging and antioxidant potential of LJEO were evaluated by a number of assays, including DPPH (1,1-diphenyl-2-picrylhydrazyl) free radical scavenging, superoxide radical scavenging, hydroxyl radical scavenging, ABTS [2,2′-azino-bis(3-ethylbenzothiazoline-6-sulphonic acid)] radical scavenging, inhibition of lipid peroxidation, and reducing power assay.

#### 3.4.1. DPPH Free Radical Scavenging Assay

The free radical scavenging potential of LJEO was evaluated using the DPPH free radical scavenging assay described by Braca *et al.* [68], with slight modification. A total of 100 µL of the reaction mixture in a 96-well flat bottom microplate (SPL Life Sciences, Gyeonggi-do, Korea) was composed of 50 µL of 0.1 mM DPPH (Sigma Aldrich, St. Louis, MO, USA) in methanol and 50 µL of different concentrations of LJEO (100–500 µg/mL). The reaction mixture was then incubated for 30 min in darkness at 37 °C with continuous shaking at 150 rpm. A sample composed of 50 µL of methanol with 50 µL of 0.1 mM DPPH was taken as a control, while 50 µL of butylated hydroxytoluene (BHT) at the concentration of 10–50 µg/mL was taken as the reference standard. The absorbance of the reaction mixture was recorded at 517 nm using a microplate reader (Infinite 200 PRO, Tecan, Mannedorf, Switzerland), and the scavenging activity was calculated as follows:

(1) $$\text{\% Scavenging=}\frac{{\text{A}}_{\left(\text{Control}\right)}–{\text{ A}}_{\left(\text{Treatment}\right)}}{{\text{A}}_{\left(\text{Control}\right)}}\text{× 100}$$

where, A_(Control)_ is the absorbance of the control and A_(Treatment)_ is the absorbance of the treatment.

#### 3.4.2. Superoxide Radical Scavenging Assay

The radical scavenging potential of LJEO was further evaluated in terms of its superoxide anion scavenging potential, which was measured using the standard procedure described by Fontana *et al.* [69]. The assay was based on a reduction reaction between nitroblue tetrazolium (NBT) and NADH in the presence of phenazine methosulfate (PMS). The reaction mixture consisted of 40 µL of 0.02 M phosphate buffer (pH 7.4), 10 µL of 15 µM PMS, 10 µL of 50 µM NBT, 10 µL of 73 µM NADH, and 30 µL of LJEO (100–500 µg/mL) or reference standard BHT (10–50 µg/mL). After all chemicals were mixed properly and incubated for 1 h at room temperature, the absorbance at 560 nm was measured against a control (reaction mixture amended with 30 µL of methanol). Scavenging activity was then analyzed using Equation (1).

#### 3.4.3. ABTS Radical Scavenging Assay

The ABTS radical scavenging activity of LJEO was measured according to the standard method described by Thaipong *et al.* [70], with slight modification. Briefly, 150 µL of reaction mixture containing 135 µL of ABTS solution (7.4 mM ABTS and 2.6 mM potassium persulfate at a 1:1 ratio and kept for 12 h in darkness) and 15 µL of different concentrations of LJEO (100–500 µg/mL) or the reference standard BHT (10–50 µg/mL) were mixed thoroughly and incubated for 2 h at 37 °C in darkness. After the incubation, the absorbance of the reaction mixture was measured at 750 nm. Reaction mixture amended with 15 µL of methanol was taken as the control. IC_50_ values (concentration of LJEO required to scavenge 50% of the ABTS radicals) were calculated by regression analysis.

#### 3.4.4. Hydroxyl Radical Scavenging Assay

The hydroxyl radical scavenging potential of LJEO was evaluated using the 2-deoxyribose oxidative degradation method described by Lopes *et al.* [71]. The reaction mixture consisted of 40 µL of mM 2-deoxyribose, 40 µL of 0.1 mM ethylenediamine-tetra acetic acid, 40 µL of 0.1 mM ferric chloride, 40 µL of 2 mM hydrogen peroxide, and 40 µL of 0.1 mM ascorbic acid prepared in a 20 mM potassium phosphate buffer (pH 7.4) and mixed with 40 µL of various concentrations of LJEO (100–500 µg/mL) or BHT (10–50 µg/mL). The mixture was incubated at 37 °C for 45 min, after which 40 µL of 2.8% TCA and 40 µL of 0.5% TBA in 0.025 M sodium hydroxide solution were added to the reaction mixture and the samples were further incubated at 90 °C for 15 min. Next, the samples were cooled and the absorbance of the reaction mixture was measured at 530 nm. A reaction mixture amended with 40 µL of methanol was taken as a control. The results are presented as the IC_50_ values (concentration of LJEO required to scavenge 50% of hydroxyl radicals) from the regression analysis.

#### 3.4.5. Inhibition of Lipid Peroxidation

The inhibitory effect of LJEO on lipid peroxidation was determined according to the standard method of non-enzymatic lipid peroxidation [72], with minor modification. The total volume of the reaction mixture (100 µL) consisted of 50 µL of bovine brain phospholipids (5 mg/mL), 10 µL of 1 mM ascorbic acid in 20 mM phosphate buffer, 10 µL of 1 mM FeCl_3_, and 30 µL of LJEO (100–500 µg/mL) or BHT (10–50 µg/mL). After being mixed thoroughly, the reaction mixture was incubated for 60 min at 37 °C, added with 100 µL of 1% TBA and, 100 µL of 30% TCA acid, and 10 µL of 4% BHT and boiled for 20 min in a boiling water bath. The sample was then cooled to room temperature, after which the absorbance was recorded at 532 nm. A reaction mixture amended with 30 µL of methanol was taken as the control. The results are presented as the IC_50_ values (concentration of LJEO required to inhibit 50% of lipid peroxidation) calculated by regression analysis.

#### 3.4.6. Reducing Power Assay

The reducing power of LJEO was determined using the standard method described by Sun *et al.* [73]. The total volume of the reaction mixture (150 µL) consisted of 50 µL of 0.2 M phosphate buffer (pH 6.6), 50 µL of 1% potassium ferricyanide, and 50 µL of LJEO (100–500 µg/mL) or BHT (10–50 µg/mL). The mixture was incubated for 20 min at 50 °C in darkness, after which the reaction was terminated by the addition of 50 µL of 10% TCA and centrifuged at 3000 rpm for 10 min. Subsequently, 50 µL of the supernatant was mixed with 50 µL of distilled water and 10 µL of 0.1% FeCl_3_ solution and incubated for another 10 min at room temperature, after which the absorbance at 700 nm was read. The results were expressed as the IC_0.5_ values (concentration of LJEO required to get 0.5 O.D. value) calculated by regression analysis.

#### 3.4.7. Total Phenolic Content

The total phenolic content in LJEO was determined according to the standard Folin–Ciocalteu phenol method described by Kujala *et al.* [74] in 100 µL of reaction mixture composed of 50 µL LJEO (0.1 mg/mL) and 50 µL 50% Folin–Ciocalteu reagent. Both solutions were mixed and incubated for 5 min at 25 °C in darkness. After incubation, 100 µL of 20% Na_2_CO_3_ solution was slowly added to the reaction mixture and incubated again for 20 min at 25 °C in darkness. Finally, the absorbance of the solution was measured at 730 nm and the phenolic content was calculated based on comparison to a standard calibration curve generated from the reference compound, gallic acid (5–50 µg/mL).

### 3.5. Statistical Analysis

All data are presented as the means ± standard deviation (SD). Statistical interpretation of the results was conducted by one-way analysis of variance (ANOVA) followed by Duncan’s test at *p* < 0.05 using the Statistical Analysis Software (SAS) (Version: SAS 9.4, SAS Institute Inc., Cary, NC, USA).

## 4. Conclusions

LJEO from the edible seaweed *L. japonica* was determined to possess high levels of fatty acid compounds with strong antioxidant and antibacterial potential, leading to its ecofriendly and beneficial utilization. LJEO contained strong antibacterial, free radical scavenging, and antioxidant potential, which might have been due to the presence of high contents of fatty acids such as tetradecanoic acid, hexadecanoic acid, (9*Z*)-hexadec-9-enoic acid, and (9*Z*,12*Z*)-9,12-Octadecadienoic acid. These properties of LJEO from edible and economic resources can make it a potential candidate for various applications in the food, pharmaceutical, and cosmetics industries.
